# Dioxin receptor regulates aldehyde dehydrogenase to block melanoma tumorigenesis and metastasis

**DOI:** 10.1186/s12943-015-0419-9

**Published:** 2015-08-05

**Authors:** María Contador-Troca, Alberto Alvarez-Barrientos, Jaime M. Merino, Antonio Morales-Hernández, María I. Rodríguez, Javier Rey-Barroso, Eva Barrasa, María I. Cerezo-Guisado, Inmaculada Catalina-Fernández, Javier Sáenz-Santamaría, Francisco J. Oliver, Pedro M. Fernandez-Salguero

**Affiliations:** Departamento de Bioquímica y Biología Molecular, Facultad de Ciencias, 06071 Badajoz, Spain; Servicio de Técnicas Aplicadas a las Biociencias, Universidad de Extremadura, 06071 Badajoz, Spain; Instituto de Parasitología y Biomedicina López Neyra, CSIC, 18016 Granada, Spain; Servicio de Anatomía Patológica, Hospital Universitario Infanta Cristina, 06071 Badajoz, Spain

**Keywords:** Dioxin receptor, Aldehyde dehydrogenase, Tumorigenesis, Lung metastasis, Cancer stem cells, Invasion

## Abstract

**Background:**

The dioxin (AhR) receptor can have oncogenic or tumor suppressor activities depending on the phenotype of the target cell. We have shown that AhR knockdown promotes melanoma primary tumorigenesis and lung metastasis in the mouse and that human metastatic melanomas had reduced AhR levels with respect to benign nevi.

**Methods:**

Mouse melanoma B16F10 cells were engineered by retroviral transduction to stably downregulate AhR expression, Aldh1a1 expression or both. They were characterized for Aldh1a1 activity, stem cell markers and migration and invasion in vitro. Their tumorigenicity in vivo was analyzed using xenografts and lung metastasis assays as well as in vivo imaging.

**Results:**

Depletion of aldehyde dehydrogenase 1a1 (Aldh1a1) impairs the pro-tumorigenic and pro-metastatic advantage of melanoma cells lacking AhR expression (sh-AhR). Thus, Aldh1a1 knockdown in sh-AhR cells (sh-AhR + sh-Aldh1a1) diminished their migration and invasion potentials and blocked tumor growth and metastasis to the lungs in immunocompetent *AhR+/+* recipient mice. However, Aldh1a1 downmodulation in AhR-expressing B16F10 cells did not significantly affect tumor growth in vivo. Aldh1a1 knockdown reduced the high levels of CD133^+^/CD29^+^/CD44^+^ cells, melanosphere size and the expression of the pluripotency marker Sox2 in sh-AhR cells. Interestingly, Sox2 increased Aldh1a1 expression in sh-AhR but not in sh-AhR + sh-Aldh1a1 cells, suggesting that Aldh1a1 and Sox2 may be co-regulated in melanoma cells. In vivo imaging revealed that mice inoculated with AhR + Aldh1a1 knockdown cells had reduced tumor burden and enhanced survival than those receiving Aldh1a1-expressing sh-AhR cells.

**Conclusions:**

Aldh1a1 overactivation in an AhR-deficient background enhances melanoma progression. Since AhR may antagonize the protumoral effects of Aldh1a1, the AhR^low^-Aldh1a1^high^ phenotype could be indicative of bad outcome in melanoma.

**Electronic supplementary material:**

The online version of this article (doi:10.1186/s12943-015-0419-9) contains supplementary material, which is available to authorized users.

## Background

Melanomas are steedely increasing in the human population often resulting in a metastatic disease with poor patient survival [1, 2]. Despite the adverse prognosis of melanoma, only a small number of molecular markers including activating mutations in the *B-RAF* [3] and *Melan-A/MART1* [4, 5] genes have been suggested as potentially relevant for the clinic. Aldehyde dehydrogenases (Aldh) are enzymes responsible for intracellular aldehyde metabolism [6] that have gained recent interest as potential diagnostic markers in melanoma. The Aldh1a1 isoform, which metabolizes retinal to retinoic acid, appears particularly important because of its ability to regulate melanogenesis [7]. Aldh1a1 has been associated to the cancer stem/tumor initiating cell phenotype in human sarcomas [8], nasopharylgeal carcinomas [9], breast carcinomas [10] and melanoma [11–13], and its level of expression and/or activity could represent a potential tool to identify stem-like cells in melanoma tumors [11, 14]. In vivo xenografts of Aldh1a1^high^ human melanoma cells in immunodeficient nude [15, 16], NGS [11] or NOD/SCID [12] mice produced larger a more aggressive tumors, suggesting that Aldh1a1 activity favoured tumorigenesis. Nevertheless, the molecular mechanisms by which Aldh1a1 influences melanoma progression are mostly unknown.

The dioxin receptor (AhR) integrates signaling pathways controlling not only xenobiotic metabolism but also tissue and organ homeostasis [17]. AhR expression has opposite roles in tumor progression increasing the growth of liver [18] and stomach tumors [19] while inhibiting intestinal carcinogenesis [20] in mice. In addition, AhR blocked the epithelial-to-mesenchymal transition (EMT) associated to tumor invasion [21] and its levels were reduced by promoter hypermethylation in acute lymphoblastic leukemia cells [22].

AhR has a role in melanoma primary tumorigenesis and lung metastasis. Indeed, we have recently reported that stable AhR knockdown in B16F10 melanoma cells enhanced their tumorigenicity and their metastatic potential to the lungs whereas constitutive AhR activation strongly blocked melanoma progression. AhR knockdown increased melanoma cell migration and invasion and the expression of mesenchymal markers α-smooth muscle actin and Snail. Interestingly, the pro-tumoral phenotype caused by AhR depletion in the tumor cell required AhR expression in the microenvironment as *AhR−/−* mice could not support tumor growth and metastatization of melanoma cells interfered for AhR [23]. The cell-autonomous effects of AhR depletion appeared to involve an EMT process and an increased content of cancer stem-like cells. Consistently, human melanoma cells and biopsies from melanoma patients had reduced AhR expression as compared to bening nevi [23]. Nevertheless, the molecular intermediates regulating the protumoral effects of AhR deficiency could not be determined.

In this study, we have found that Aldh1a1 upregulation is likely an intermediate factor promoting melanoma growth and metastasis in AhR depleted cells. Consistent with that hypothesis, AhR knockdown failed to exert a pro-tumoral effect when Aldh1a1 was simultaneously inactivated. Interestingly, depletion of basal Aldh1a1 levels in AhR-expressing melanoma cells did not significantly affect tumor growth, suggesting that the overactivation of Aldh1a1 is likely a causal factor increasing the tumorigenicity of AhR deficient melanoma cells. Therefore, the tumor suppresor role of AhR in melanoma [23] could take place by antagonizing the Aldh1a1 activity. We suggest that the coordinated expression of AhR and Aldh1a1 could be a useful molecular marker in melanoma.

## Results

### AhR levels inversely correlated with Aldh1a1 expression in melanoma cells: AhR knockdown increased Aldh1a1 activity

**Fig. 1:**
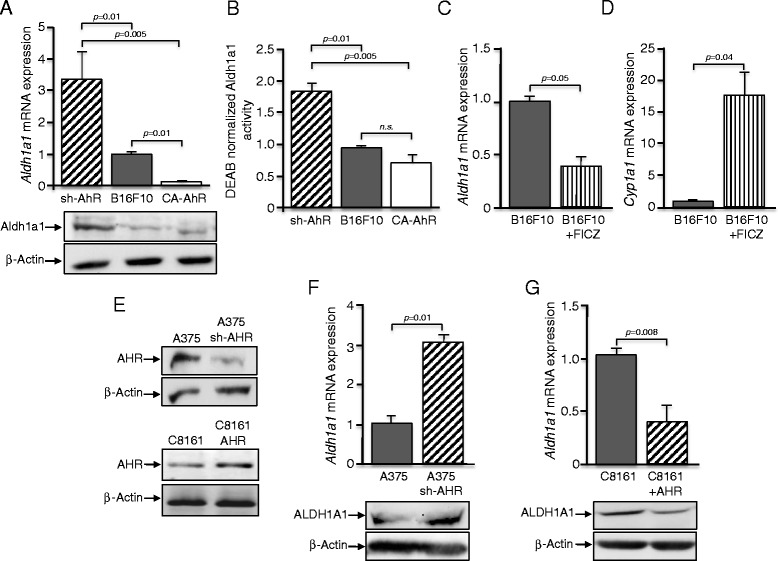
AhR expression represses Aldh1a1 in mouse and human melanoma cells. **a** B16F10-derived cell lines were previously generated to stably express very low (sh-AhR), basal (B16F10) or a constitutively active (CA-AhR) AhR [23]. They were analyzed for *Aldh1a1* mRNA expression by RT-qPCR and for protein expression by immunoblotting. **b** Aldh1a1 activity was measured by flow cytometry in sh-AhR, B16F10 and CA-AhR cells using the Aldefluor reagent kit. To normalize Aldh1a1 activity, measurements were taken in presence and absence of the Aldh1-specific inhibitor diethylamino-benzaldehyde (DEAB). Values were then referred to those obtained for wild type B16F10 cells. **c**, **d** B16F10 cells were treated with the AhR endogenous ligand FICZ (5 nM) and the mRNA levels of *Aldh1a1* (**c**) and *Cyp1a1* (**d**) determined by RT-qPCR. **e** A375 human melanoma cells were transfected with a sh-AHR to downmodulate their high basal receptor levels (upper). C8161 human melanoma cells were transfected with an AHR expression vector (AHR) to increase their low receptor levels. **f**, **g** *ALDH1A1* mRNA and protein amounts were determined in A375 (**f**) and C8161 (**g**) cell lines by RT-qPCR and immunoblotting, respectively. The expression of β-Actin was used to normalize protein loading. RT-qPCR data were normalized by *Gapdh* expression and represented as 2^-ΔΔCt^. The B16F10 cell line was also transduced with a retrovirus containing the empty vectors used to produce the sh-AhR and CA-AhR lines. Determinations were done in triplicate in at least two different cell cultures. Data are shown as mean ± SD

We have shown that stable AhR knockdown (sh-AhR) increases primary tumorigenesis and lung metastasis of mouse melanoma cells and that AhR expression was reduced in advanced human melanomas [23]. The increased tumorigenic potential of sh-AhR melanoma cells correlated with higher levels of cancer stem-like markers, suggesting a more undifferentiated status [23]. On the other hand, aldehyde dehydrogenase 1a1 (Aldh1a1) has been recently identified as a potential melanoma promoter and a regulator of the cancer stem cell phenotype [11–13, 24]. Here, we have investigated the contribution of Aldh1a1 to the pro-tumorigenic effects associated to AhR deficiency. AhR knockdown in mouse melanoma B16F10 cells significantly increased *Aldh1a1* mRNA and protein expression as compared to wild type B16F10 cells (Fig. 1a). In contrast, B16F10 cells expressing a constitutively active receptor (CA-AhR) had a significant reduction in *Aldh1a1* mRNA and protein levels as compared to sh-AhR cells (Fig. 1a). Aldh1a1 protein levels did not significantly vary between wild type B16F10 and CA-AhR cells, regardless of their differences in mRNA expression. Based on these results, we next analyzed Aldh1a1 activity in our cell lines using the Aldefluor assay. To normalize for any potential Aldh-independent activity, determinations were done in presence and absence of the Aldh1-specific inhibitor diethylamino-benzaldehyde (DEAB) [8, 25]. We found that Aldh1a1 activity was significantly higher in sh-AhR than in wild type and CA-AhR B16F10 cells, and that constitutive AhR activation did not affect basal Aldh1a1 activity (Fig. 1b). Aldehyde dehydrogenase activity, as measured by the Aldefluor assay, results from the sum of various isoenzymes, particularly Aldh1a1 and Aldh1a3 [26]. However, since *Aldh1a3* expression was not significantly different between sh-AhR, B16F10 and CA-AhR cells (Additional file 1: Figure S1A), we considered the differences obtained in aldehyde dehydrogenase activity as mostly coming from Aldh1a1. To further analyze the inhibitory role of AhR on *Aldh1a1* expression, B16F10 cells were treated with the tryptophan metabolite and bone-fide AhR endogenous ligand 6-formylindolo[3,2-b]carbazole (FICZ) [27, 28]. The results showed that FICZ significantly reduced *Aldh1a1* mRNA levels in B16F10 cells (Fig. 1c). Control experiments confirmed that FICZ was an efficient AhR agonist in B16F10 cells as shown by the induction of its target gene *Cyp1a1* (Fig. 1d).

We then decided to analyze if *Aldh1a1* was a direct transcriptional target of AhR. Sequence analysis revealed that the upstream promoter region of the mouse *Aldh1a1* gene has four xenobiotic responsive elements (XRE, 5′-GCGTG- 3′) located at positions −265, −726, −3114 and −3158 with respect to the transcription start site. Chromatin immunoprecipitation (ChIP) was used to detect AhR binding to these XRE sites essentially as described [29–31]. However, we could not demonstrate direct binding of AhR to those XRE sites even under modified crosslinking conditions that favour protein-DNA interactions by the addition of DSG (disuccinimidyl glutarate) of DMA (dimethyl apidimate) [32] (Additional file 1: Figure S1B). Thus, at present, we cannot confirm whether or not AhR directly binds to the mouse *Aldh1a1* gene promoter.

?A3B2 tlsb=-.15pt?>The inverse correlation between AhR and Aldh1a1 in mouse melanoma cells was also observed in human melanoma cells previously reported to express high (A375) or low (C8161) receptor levels [23]. AHR downmodulation by a sh-AHR in A375 cells (Fig. 1e) produced a significant increase in their basal *ALDH1A1* mRNA and protein levels (Fig. 1f). By contrast, AHR over-expression in C8161 cells (AHR) (Fig. 1e) markedly reduced *ALDH1A1* mRNA and protein amounts as compared with basal C8161 cells (Fig. 1g). Thus, AhR and Aldh1a1 had opposite expression patterns in both murine and human melanoma cells.

### Aldh1a1 knockdown blocked the increase in migration and invasion produced by AhR depletion

**Fig. 2:**
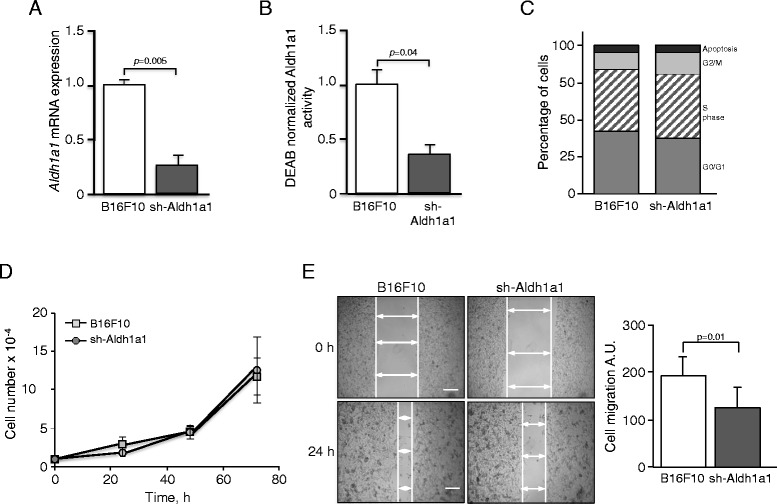
Aldh1a1 expression and activity can be downmodulated in B16F10 melanoma cells. Wild type B16F10 melanoma cells were retrovirally transduced with a sh-RNA molecule as indicated in the Methods to produce the sh-Aldh1a1 cell line. **a** *Aldh1a1* mRNA levels were determined in wild type B16F10 and sh-Aldh1a1 cells by RT-qPCR. **b** Aldh1a1 activity was quantified by flow cytometry in both cells lines using the Aldefluor reagent kit. **c** B16F10 and sh-Aldh1a1 cells were analyzed for cell cycle distribution by flow cytometry. The percentage of cells in each cell cycle phase is indicated. **d** The kinetics of cell proliferation were determined by counting de number of cells during a 72 h period. **e** The migration of B16F10 and sh-Aldh1a1 cells was analyzed using wound healing assays. The B16F10 cell line was also transduced with a retrovirus containing the empty vectors used to produce the sh-Aldh1a1 cell line. Measurements were done in triplicate in two different cultures. At least 6 fields were analyzed for each wound in panel (**e**). Data are shown as mean ± SD. A.U. stands for arbitrary units

We then hypothesized that the increased tumorigenic and metastatic potential of sh-AhR melanoma cells [23] could depend, at least in part, to their high Aldh1a1 activity. To analyze such possibility, we first used retroviral transduction to interfere Aldh1a1 expression in B16F10 cells (sh-Aldh1a1). sh-Aldh1a1 cells had a significant reduction in *Aldh1a1* mRNA levels (Fig. 2a) and Aldh1a1 activity (Fig. 2b) with respect to wild type B16F10 cells. Cell counting over time and flow cytometry analysis of the different phases of the cell cycle revealed that stable Aldh1a1 downmodulation did not significantly affect cell cycle distribution (Fig. 2c) or proliferation rates (Fig. 2d). However, wound-healing assays revealed that Aldh1a1 interference reduced the migration ability of B16F10 melanoma cells (Fig. 2e).

**Fig. 3:**
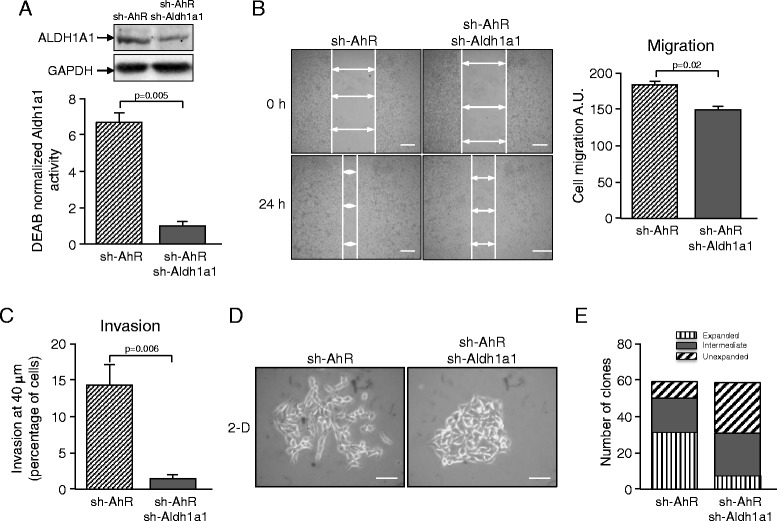
Aldh1a1 knockdown reduces the migratory and invasive phenotypes produced by AhR depletion. **a** sh-AhR B16F10 melanoma cells were stably transduced with a sh-RNA for *Aldh1a1* to generate the sh-AhR + sh-Aldh1a1 cell line. sh-AhR and sh-AhR + sh-Aldh1a1 cells were analyzed for Aldh1a1 protein expression by immunoblotting and for Aldh1a1 activity by flow cytometry using the Aldefluor reagent kit. To normalize Aldh1a1 activity, measurements were taken in presence and absence of the Aldh1-specific inhibitor diethylamino-benzaldehyde (DEAB). Values were then referred to those obtained for sh-AhR + sh-Aldh1a1 cells. **b** sh-AhR and sh-AhR + sh-Aldh1a1 cells were grown to confluence and then used for wound healing migration assays. **c** Both cell lines were cultured in matrigel transwells and analyzed for their invasive potential by confocal microscopy. Cell invasion at a depth of 40 μm is shown. **d** Clone formation in 2-D was determined by growing sh-AhR and sh-AhR + sh-Aldh1a1 cells at low cell density in plain tissue culture dishes. Clones were counted as unexpanded (compact clones with a dense cellularity), expanded (cells spreading out with reduced cell-cell interactions) or intermediate clones. **e** Clones were photographed, counted and their spreading analyzed using the ImageJ software. Three levels of spreading were established for compact (unexpanded), intermediate and fully expanded clones. Bars correspond to 100 μm (**b**) and 50 μm (**d**). A.U. arbitrary units. Experiments were done in duplicate or triplicate in three different cultures. At least 6 fields were analyzed for each wound in panel B. Data are shown as mean ± SD. A.U. stands for arbitrary units

As Aldh1a1 expression and activity could be manipulated in B16F10 cells, and since we sought to investigate the extent to which Aldh1a1 contributes to the increased tumorigenicity caused by AhR knockdown [23], we decided to use retroviral transduction to deplete Aldh1a1 in AhR-interfered B16F10 cells. The cell line thus generated (hereafter sh-AhR + sh-Aldh1a1) was used to analyze the effects of Aldh1a1 expression in an AhR deficient background. Control experiments revealed that sh-AhR + sh-Aldh1a1 cells had a significant reduction in both Aldh1a1 protein expression and Aldh1a1 activity as compared to sh-AhR cells (Fig. 3a). Aldh1a1 depletion moderately blocked the migration of sh-AhR + sh-Aldh1a1 cells in wound healing assays with respect to Aldh1a1 expressing sh-AhR cells (Fig. 3b). Confocal microscopy analyses of matrigel-coated culture transwells showed that sh-AhR + sh-Aldh1a1 cells had a significant impairment to invade as compared to sh-AhR melanoma cells (Fig. 3c). Both cell lines were grown at low density in 2-D cultures in order to determine their clonogenicity potential. A qualitative scale was established to account for the appearance of unexpanded (compact clones with a dense cellularity), expanded (clones in which cells spread out and had reduced cell-cell interactions) or intermediate clones. We found that sh-AhR + sh-Aldh1a1 cells formed compact clones with enhanced cell-cell interactions and with a less invasive phenotype than that exhibited by sh-AhR cells (Fig. 3d). Consistently, sh-AhR + sh-Aldh1a1 cells formed a fewer number of fully expanded clones than sh-AhR cells under the same culturing conditions (Fig. 3e). Thus, the pro-migratory and pro-invasive phenotypes observed in AhR knockdown cells may be in part dependent on Aldh1a1 activity.

### Aldh1a1 was involved in maintaining the subpopulation of cancer stem-like cells

**Fig. 4:**
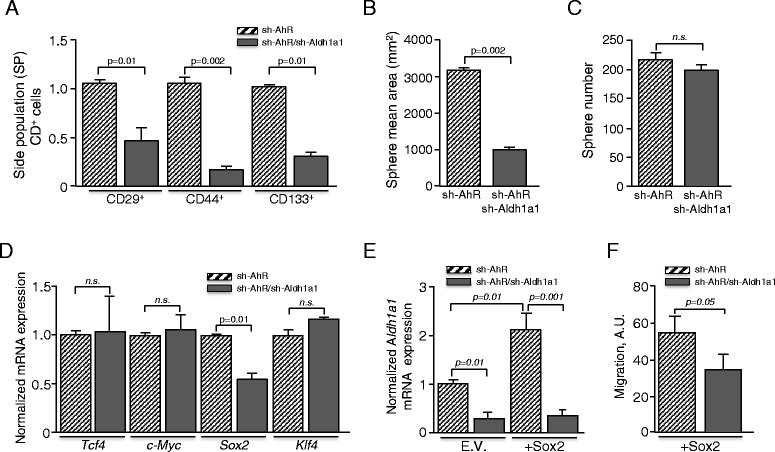
Aldh1a1 downmodulation reduces cancer stem-like and pluripotency markers in AhR knockdown melanoma cells. **a** sh-AhR and sh-AhR + sh-Aldh1a1 cells were stained for CD133^+^ (CD133-PE), CD44^+^ (CD44-PerCP) and CD29^+^ (CD29-FITC) and analyzed by flow cytometry. **b**, **c** The same number of sh-AhR and sh-AhR + sh-Aldh1a1 cells was grown in ultra-low adherence culture dishes. The size (**b**) and number (**c**) of the melanospheres formed were analyzed using a Cell-R microscope and the ImageJ software. **d** The mRNA expression levels of the pluripotency genes *Sox2*, *Klf4*, *c-Myc* and *Tcf4* were analyzed by RT-qPCR using total RNA isolated from sh-AhR and sh-AhR + sh-Aldh1a1 cells. **e** sh-AhR and sh-AhR + sh-Aldh1a1 cells were transiently transfected with a Sox2 expression vector and the mRNA levels of *Aldh1a1* quantified by RT-qPCR. The empty vector (E.V.) was also transfected to normalize gene expression. **f** sh-AhR and sh-AhR + sh-Aldh1a1 cells were transfected with a Sox2 expression vector and their migration potential determined in wound healing assays. RT-qPCR data were normalized by *Gapdh* expression and represented as 2^-ΔΔCt^. A.U. arbitrary units. Measurements were done in triplicate in two different cultures of each cell line. At least 6 fields were analyzed for each wound in panel (**f**). Data are shown as mean ± SD. A.U. stands for arbitrary units. *n.s.* = statistically non-significant

The tumorigenic potential of sh-AhR melanoma cells was found to be associated with an increase in the pool of cancer stem-like cells [23]. The effects of Aldh1a1 in maintaining melanoma progression through the control of cancer stem cells have been almost exclusively studied using xenografts of human cell lines in immunodeficient mice [11, 12, 14–16]. We next addressed whether Aldh1a1 was required to maintain the population of stem-like cells in our melanoma cell cultures. We first used flow cytometry to isolate the side population (SP) cells present in sh-AhR and sh-AhR + sh-Aldh1a1 cultures based on their ability to activate the ABCG2 efflux pump [33]. SP cells from each cell line were then further analyzed for the expression of candidate stem cell markers such as CD133^+^, whose upregulation has been associated to increased clonogenic capacity and tumorigenicity in melanoma [34–37]. We found that sh-AhR + sh-Aldh1a1 SP-cells had a significant reduction in the number of CD133^+^/CD29^+^/CD44^+^ cells with respect to sh-AhR SP-cells (Fig. 4a). Sphere formation assays under ultra-low attachment conditions showed that sh-AhR + sh-Aldh1a1 cells produced melanospheres of smaller size and containing a fewer number of undifferentiated cells than those originated from sh-AhR cells (Fig. 4b). However, the total number of spheres generated by sh-AhR cells was not significantly altered by Aldh1a1 depletion (Fig. 4c). mRNA levels of differentiation related genes *Sox2*, *Klf4*, *c-Myc* and *Tcf4* showed no significant differences between sh-AhR and sh-AhR + sh-Aldh1a1 cells, except for *Sox2*, which was downmodulated in sh-AhR + sh-Aldh1a1 cells (Fig. 4d). This apparent correlation between *Sox2* and *Aldh1a1* was supported by the observation that ectopic Sox2 expression increased *Aldh1a1* mRNA levels in sh-AhR but not in Aldh1a1-interfered melanoma cells (Fig. 4e), indicating that Sox2 could play a role in maintaining Aldh1a1 expression. Moreover, Sox2 failed to rescue the migration of Aldh1a1-depleted sh-AhR + sh-Aldh1a1 cells (Fig. 4f), further supporting a functional interaction between Aldh1a1 and Sox2 in murine melanoma cells.

### Aldh1a1 depletion impaired primary tumorigenesis and lung metastasis of AhR knockdown melanoma cells

**Fig. 5:**
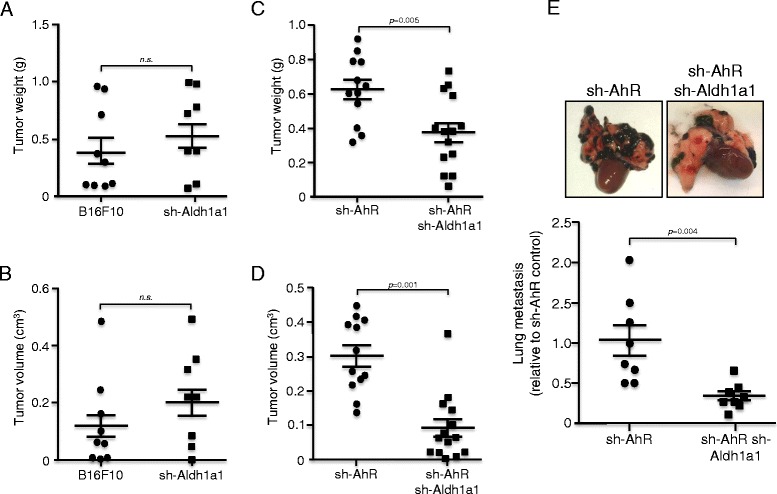
Aldh1a1 knockdown blocks primary tumorigenesis and lung metastasis induced by AhR depletion. **a**, **b** B16F10 and sh-Aldh1a1 cells were injected subcutaneously in the dorsal flank of *AhR+/+* immunocompetent mice. Each cell line was injected in one of the two flanks of each mouse. Tumors were collected, weighted (**a**) and their volume (**b**) calculated using the formula [length x width^2^ x 0.4]. A total of 9 and 8 tumors were analyzed for B16F10 and sh-Aldh1a1 cells, respectively. **c**, **d** sh-AhR and sh-AhR + sh-Aldh1a1 cells were injected subcutaneously in the dorsal flank of *AhR+/+* immunocompetent mice. Each cell line was injected in one of the two flanks of each mouse. Tumors were collected, weighted (**c**) and their volume (**d**) calculated using the formula [length × width^2^ × 0.4]. A total of 12 and 14 tumors were analyzed for sh-AhR and sh-AhR + sh-Aldh1a1 cells, respectively. **e** For the analysis of metastasis, both cell lines were inoculated through the tail vein in the same strain of recipient mice indicated above. Lungs were removed and the number of metastatic nodules counted and represented. Eight mice were injected for each cell line. The B16F10 cell line was also transduced with a retrovirus containing the empty vectors used to produce the sh-Aldh1a1 cell line. Data are shown as mean ± SD. *n.s.* = statistically non-significant

We then decided to investigate whether Aldh1a1 depletion affected primary tumorigenesis and metastasis of AhR knockdown B16F10 cells. We first examined if single Aldh1a1 knockdown affected tumor formation in AhR expressing cells. To do that, B16F10 wild type and sh-Aldh1a1 cells were inoculated in either dorsal flank of *AhR+/+* immunocompetent mice. Tumor analysis revealed that sh-Aldh1a1 cells produced tumors of similar weight (Fig. 5a) and volume (Fig. 5b) than those originated from wild type B16F10 cells, indicating that, at least in our experimental conditions using immunocompetent recipient mice, Aldh1a1 depletion alone was not enough to affect melanoma primary tumorigenesis. We next performed similar experiments injecting sh-AhR and sh-AhR + sh-Aldh1a1 cells in either dorsal flank of *AhR+/+* immunocompetent mice. AhR + Aldh1a1 depletion produced tumors of significantly smaller weight (Fig. 5c) and volume (Fig. 5d) than those generated by Aldh1a1-expressing sh-AhR cells. When injected through the tail vein, sh-AhR + sh-Aldh1a1 cells exhibited a close to three fold reduction in their metastatic potential to the lungs as compared to sh-AhR cells (Fig. 5e).

**Fig. 6:**
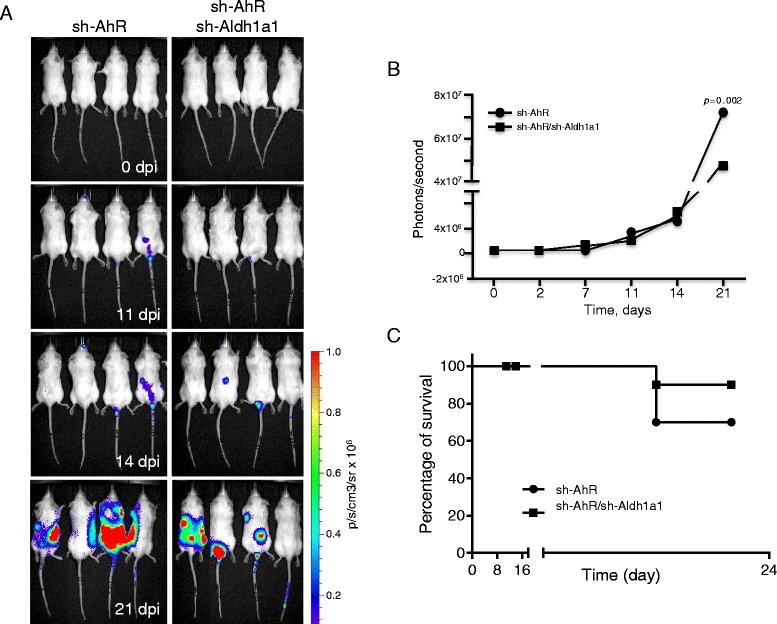
Reduced Aldh1a1 expression blocks the metastatic burden of AhR depletion in melanoma cells. **a** sh-AhR:LUX and sh-AhR + sh-Aldh1a1:LUX cells, stably expressing the luciferase protein, were injected through the tail vein into C57BL6 albino mice. Mice were injected i.p. with luciferin at the time of cell inoculation and after 2, 7, 11, 14 and 21 days and analyzed using an IVIS equipment. **b** Metastatic dissemination was quantified by measuring light emission and the results have been represented as photons/second for each cell line. **c** Kaplan-Meier plot for the survival of mice injected with sh-AhR:LUX or sh-AhR + sh-Aldh1a1:LUX cells. A total of 10 C57BL6 albino mice were used for each experimental condition. Data are shown as mean ± SD

To further analyze the effects of Aldh1a1 in the metastatic burden of melanoma cells, we used retroviral transduction to produce sh-AhR-Luc and sh-AhR + sh-Aldh1a1-Luc cells expressing the in vivo marker luciferase. In vivo imaging analyses (IVIS) revealed that metastatic dissemination started 11 days after inoculation for both cell lines (Fig. 6a). At later times (e.g., 21 days), total metastatic load was reduced in sh-AhR + sh-Aldh1a1 cells with respect to sh-AhR cells (Fig. 6b). Metastatic dissemination correlated to a certain degree with mice survival, which was moderately extended in mice receiving sh-AhR + sh-Aldh1a1 cells (Fig. 6c). These results suggested that Aldh1a1 is a likely intermediate in the tumor promoting effects caused by AhR depletion.

**Fig. 7:**
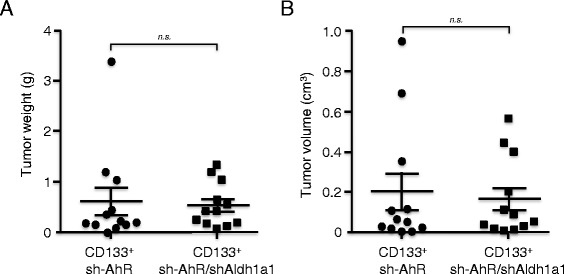
Aldh1a1 depletion does not significantly affect the intrinsic cancer stem-like phenotype of melanoma cells. **a**, **b** The same number of viable CD133^*+*^ cells were isolated from each line by cell-sorting and injected into the dorsal flanks of *AhR+/+* recipient mice. Each cell line was injected in one of the two flanks of each mouse. After 15 days, tumors were harvested, weighted (**a**) and their volume (**b**) calculated using the formula [length × width^2^ × 0.4]. A total of 12 tumors were analyzed for each cell line. *n.s.* = statistically non-significant

Aldh1a1 knockdown in sh-AhR + sh-Aldh1a1 cells produced melanospheres and tumors of significantly smaller size than those arising from sh-AhR cells. Such diminished tumorigenicity could be due to a reduction in the number of cancer stem-like cells present in sh-AhR + sh-Aldh1a1 cultures and/or to a less undifferentiated status. To address this question, we used flow cytometry-assisted cell sorting to isolate viable CD133^+^ cells from the SP subpopulations of sh-AhR and sh-AhR + sh-Aldh1a1 cultures. The same number of CD133^+^ cells from each cell line was then subcutaneously injected into either flank of *AhR+/+* immunocompetent recipient mice. We found that tumors formed by sh-AhR + sh-Aldh1a1 CD133^+^ cells were similar in weight (Fig. 7a) and volume (Fig. 7b) than those produced by sh-AhR CD133^+^ cells, suggesting that a reduction in the number of cancer stem-like cells could be the most likely factor compromising tumor growth by Aldh1a1 depleted AhR + sh-Aldh1a1 cells.

## Discussion

Defining the mechanisms and the molecular intermediates that regulate growth and metastatic dissemination of tumor cells represents a major topic in cancer. We have previously reported that stable AhR knockdown increased the tumorigenic and metastatic abilities of B16F10 melanoma cells, suggesting that this receptor could have a tumor suppressor role in melanoma. Interestingly, the increased tumorigenicity of sh-AhR cells appeared to be associated to the accumulation of cancer stem-like cells [23]. In this work, we have investigated the Aldh1a1 enzyme as a molecular intermediate whose upregulation in AhR depleted cells could promote melanoma progression. The main findings from this study are that AhR expression restrains Aldh1a1 activity in murine melanoma cells and that, as a consequence, AhR depletion results in Aldh1a1 upregulation and in the exacerbation of melanoma primary tumorigenesis and metastasis in vivo. In agreement with these findings, stable Aldh1a1 knockdown was able to block the effects induced by AhR deficiency both in vitro and in vivo. We therefore suggest that AhR may block melanoma by inhibiting the pro-tumoral effects of Aldh1a1, and that coordinated expression of AhR and Aldh1a1 could be a useful molecular marker in melanoma.

It is generally accepted that Aldh1a1 is a pro-tumoral enzyme associated to poor prognosis in several cancer types. Aldh1a1 appears to have a causal role in maintaining the undifferentiated status of breast [10, 38], lung [39] and prostate [40] tumors and of soft tissue sarcomas [8], carcinomas [9, 41] and melanoma. Regarding melanoma, this enzyme has been suggested as a marker to identify subpopulations of cancer stem cells [11, 12]. We, and others, have also established an association between AhR and melanoma. AhR mediates growth inhibition of melanoma cells by the therapeutic drug leflunomide [42], whereas constitutive AhR activation repressed melanoma growth and metastasis [23]. The results presented here, propose the existence of regulatory pathway between AhR and Aldh1a1 in melanoma considering that the pro-tumorigenic effects of Aldh1a1 overexpression were significantly augmented in an AhR deficient background. The fact that a constitutively active AhR or a bona-fide endogenous ligand could repress Aldh1a1 gives additional support to such pathway and suggests that non-toxic AhR ligands could inhibit the progression of melanomas having high levels of Aldh1a1 activity. Although our results strongly support that AhR modulates *Aldh1a1* mRNA expression in murine melanoma cells, it is still undefined whether it requires AhR binding to the XRE elements present in the *Aldh1a1* promoter. The lack of AhR binding in chromatin immunoprecipitation assays would suggest an indirect mechanism involving additional intermediates or, alternatively, a failure of the available reagents to detect the specific AhR-DNA complexes organized at those XRE sites. AhR has been shown to regulate xenobiotic-unrelated genes by direct promoter binding (*Vav3* oncogene [29]) or by indirect mechanisms involving changes in histone acetylation (latent TGFβ binding protein *Ltbp1* [32]).

Aldh1a1 downmodulation reduced melanoma cell migration and invasion in vitro and tumor growth and metastatic dissemination in vivo. However, these effects were only seen in cells simultaneously depleted of AhR expression. Thus, it seems that Aldh1a1 knockdown impairs the cell-autonomous potential of AhR-depleted cells to develop larger and more metastatic lesions. Since AhR negatively modulate Aldh1a1 activity, melanoma tumors could restrict AhR expression in order to maintain the tumor promoting activity of Aldh1a1. In this context, the inverse correlation observed between AhR and Aldh1a1 could gain additional interest considering that AhR protein levels were reduced in high grade human melanomas as compared to non-malignant nevi [23]. We have attempted to determine Aldh1a1 levels by immunohistochemistry in our tissue microarrays (TMA) containing human melanomas expressing different amounts of AHR [23]. However, the antibodies to detect human ALDH1A1 commercially available do not give enough sensitivity and specificity to correlate ALDH1A1 and AHR in such tissue sections. The development of clinically validated reagents could help solve this limitation.

Aldh1a1 and AhR converge at the control of the cancer stem-like phenotype in melanoma cells, although with an opposite pattern that resembles that found for tumor growth. High Aldh1a1 expression is being considered not only a marker [43, 44] but also a potential tool to isolate stem-like cells in melanoma [11–13]. Regarding AhR, its repression favours the growth of human hematopoietic stem cells [45], the development of mouse embryonic stem cells [46], the acquisition of an EMT phenotype in epithelial cells [21] and the expansion of stem-like cells in melanoma [23]. Consistent with such opposing functions of these two proteins in tumor development, our results suggest that the enhanced tumoral response produced by AhR deficient cells could be at least partially due to their increased content in cancer stem-like cells perhaps as a consequence of Aldh1a1 overactivation. Indeed, Aldh1a1 downmodulation markedly reduced the number of cancer stem-like cells and impaired primary tumorigenesis and lung metastasis in AhR depleted melanoma cells. Among the molecules that could mediate the pro-tumoral effects of Aldh1a1, the stem cell regulator Sox2 appears as a plausible candidate. The evidences showing that Sox2 expression was coincident with Aldh1a1 overactivation, and that Sox2 expression inhibited *Aldh1a1* in AhR knockdown cells, suggest the existence of a coordinated pathway involving AhR-Aldh1a1-Sox2 in the regulation of stemness in melanoma cells. In agreement with such possibility, recent studies have shown that high Aldh1a1 and Sox2 expression correlates with an undifferentiated and metastatic phenotype in oral squamous cell carcinomas [41] and that Sox2 depletion blocks the tumor-initiating ability of human melanoma cells expressing high levels of Aldh1a1 [14].

The functional interaction between Aldh1a1 and AhR could be also relevant for the control of migration and invasion of melanoma cells. Previous studies have shown that epithelial cells lacking AhR migrate faster in vitro and in vivo [47] and that AhR downmodulation induces an epithelial-to-mesenchymal transition eventually resulting in enhanced cell invasion [21]. Notably, siRNA-mediated transient silencing of ALDH1A1 inhibited cell migration in human H2087 non-small cell lung cancer cells [48] and invasion in A498 human renal carcinoma cells [49]. Although the mechanisms by which the AhR-Aldh1a1 axis participates in the control of cell migration and invasion remain largely unknown, they may involve cell-cell and cell-substratum signalling since recent work has shown that AhR regulates cytoskeleton organization and the dynamics of focal adhesions [47, 50, 51], β1 integrin activation [52] and caveolin-1 dependent signalling [53].

## Conclusions

In summary, we present evidences for the existence of a regulatory mechanism by which AhR modulates Aldh1a1 expression and activity in melanoma cells. In an AhR deficient background, increased Aldh1a1 activity likely supports primary tumorigenesis and lung metastasis of melanoma cells, whereas a reduction in Aldh1a1 activity impaired the pro-tumorigenic effects caused by AhR depletion. Since AhR expression appears to be reduced in advanced human melanomas, it will be interesting to investigate whether Aldh1a1 becomes upregulated in the same tumors and if it contributes to disease progression. It can be speculated that AhR activation by endogenous non-toxic ligands could repress Aldh1a1 expression blocking tumor growth. Therefore, the functional interaction between AhR and Aldh1a1 could be of potential interest for melanoma progression and therapy.

## Methods

### Cell lines and mice

B16F10 mouse melanoma cells were from the American Type Culture Collection (ATCC). Human melanoma C8161 and A375 cell lines were authenticated by DNA profiling using 8 different and highly polymorphic short tandem repeat (STR) loci (German Biological Resource Centre DSMZ). B16F10 cells were also analyzed for their melanin production. All melanoma cells were cultured in DMEM containing 10 % FBS, 100 U/ml penicillin, 100 μg/ml streptomycin and 2 mM L-glutamine at 37 °C and 5 % CO_2_ atmosphere. For primary tumorigenesis and lung metastasis experiments, wild type immunocompetent *AhR+/+* mice [54] at 8–10 weeks of age were inoculated with genetically modified B16F10 melanoma cells as indicated below. To analyze systemic metastatic dissemination, genetically modified B16F10 melanoma cells were injected into albino C57BL6 mice (Harlan). All the experiments involving mice were approved by the Bioethics and Biosecurity Commissions of the University of Extremadura and the Instituto de Parasitología y Biomedicina López Neyra (IPBLN, CSIC) within the approved project BFU-2011-22678 and under the approved protocol 11/2011. Mice had free access to water and rodent chow.

### Antibodies, vectors and reagents

The affinity purified AhR antibody (SA-210) was from Enzo, CD133-PE, CD44-PerCP and CD29-FITC were from Biolegend. Antibodies for Aldh1a1 were from Becton-Dickinson and Santa Cruz Biotechnology. The antibody for β-actin was obtained from Sigma-Aldrich. Matrigel-coated transwells were from Becton-Dickinson. The iScript™ Reverse Transcription Supermix was from Bio-Rad and the SYBR® Select Master Mix for real-time PCR from Life Technologies. The AhR agonist 6-formylindolo[3,2-b]carbazole (FICZ) was from Enzo and it was used at a 5 nM concentration.

### Retroviral transduction

B16F10 melanoma cells were stably transduced with retroviruses containing small hairpin RNAs for AhR (sh-AhR), Aldh1a1 (sh-Aldh1a1) or AhR + Aldh1a1 (sh-AhR + sh-Aldh1a1) essentially as described [21, 23]. To produce the sh-AhR + sh-Aldh1a1 cell line, B16F10 sh-AhR cells [23] were infected with an sh-Aldh1a1-containing retrovirus. Knockdown of Aldh1a1 was determined by real time RT-qPCR and Western blotting and by flow cytometry using the Aldefluor reagent kit as described below. In certain experiments, a B16F10 cell line engineered to express a constitutively active receptor [23] was also used. The human A375 melanoma cell line was also transduced with a retrovirus containing a human sh-AhR (A375 sh-AhR) as previously indicated [23]. The sh-RNA sequence for *AhR* was: 5′TGCTGTTGACAGTGAGCGCTCAGTGTATCTTGTAAAGAAATAGTGAAGCCACAGATGTATTTCTTTACAAGATACACTGAATGCCTACTGCCTCGGA3′. The sh-RNA sequence for *Aldh1a1* was: 5′TGCTGTTGACAGTGAGCGCAGATGCCAGGTGAAGAGCCGTTAGTGAAGCCACAGATGTATCGGCTCTTCTCCTGGCTTCTTTGCCTACTGCCTCGGA3′.

### Transient transfection

Transient transfections were done in human C8161 and mouse B16F10 melanoma cells. Briefly, 2×10^5^ cells were cultured in 35 mm dishes for 24 h. Then, cells were transfected with 1 μg of the expression vectors pBABE:AhR (C8161) or pBABE:Sox2 (B16F10) using the TurboFect reagent (Fermentas) as indicated by the manufacturer. Transfections were performed in Opti-MEM® I Reduced Serum Medium (Life Technologies). Experiments were done 24 h after transfection.

### SDS-PAGE and immunoblotting

SDS-PAGE and immunoblotting were performed using total protein extracts from A375, C8161, sh-AhR and sh-AhR + sh-Aldh1a1 cells as previously described [55].

### Reverse transcription and real-time PCR

Total RNA was isolated using the High Pure RNA Isolation Kit (Roche). Reverse transcription was performed using random priming and the iScript Reverse Transcription Super Mix (Bio-Rad). Real-time PCR was used to quantify the mRNA levels of *Aldh1a1*, *Sox2*, *Tcf4*, *c-Myc*, *Klf4* and *Cyp1a1*. Reactions were done using SYBR® Select Master Mix (Life Technologies) in a Step One Thermal Cycler (Applied Biosystems) essentially as described [52]. *Gapdh* was used to normalize gene expression (ΔCt) and 2^-ΔΔCt^ to calculate changes in mRNA levels with respect to control or untreated conditions. The following primer sequences were used to analyze murine genes: *Aldh1a1* 5′-CTCCTGGCGTGGTAAACATT-3′ (forward) and 5′-CCATGGTGTGCAAACTCAAC-3′ (reverse); *Aldh1a3* 5′-ATCAAACCCACGGTCTTCTC-3′ (forward) and 5′-TTTGTCCAGGTTTTTGGTGA-3′ (reverse); *Sox2* 5′-CACAACTCGGAGATCAGCAA-3′ (forward) and 5′-CCGGGAAGCGTGTACTTATC-3′ (reverse); *Tcf4* 5′-CTTCTTTGGCGAGTGGACA-3′(forward) and 5′-GTGACCCAAGATCCCTGCT-3′ (reverse); *Klf4* 5′-CACAAGTCCCCTCTCTCCAT-3′ (forward) and 5′-TTTCTCGCCTGTGTGAGTTC-3′ (reverse); *c-Myc* 5′-CCTGACGACGAGACCTTCA-3′ (forward) and 5′-TGGTAGGAGGCCAGCTTCT-3′ (reverse); *Cyp1a1* 5′-ACAGACAGCCTCATTGAGCA-3′ (forward) and 5′-GGCTCCACGAGATAGCAGTT-3′ (reverse); *Gapdh* 5′-TGAAGCAGGCATCTGAGGG-3′ (forward) and 5′-CGAAGGTGGAAGAGTGGGAG-3′ (reverse). For human genes, the following primers were used: *ALDH1A1* 5′-AAACGGAGGCCAGGATAACT-3′ (forward) and 5′-CCATGGTGTGCAAACTCAAC-3′ (reverse); *GAPDH* 5-CCACCCAGAAGACTGTGGAT-3′ (forward) and 5′-TTCTAGACGGCAGGTCAGGT-3′ (reverse).

### Chromatin Immunoprecipitation (ChIP)

Chromatin immunoprecipitation (ChIP) was performed essentially as described [30–32]. In brief, protein-DNA complexes were crosslinked by formaldehyde treatment and sonicated for 20 min in a Bioruptor (Dianogenode) apparatus. Following centrifugation, the sonicated DNAs were first preincubated with protein A/G agarose beads to reduce unspecific binding and then immunoprecipitated overnight at 4 °C with 4 μg of anti-AhR antibody in presence of fresh protein A/G agarose beads. DNAs were eluted from the immunoprecipitates, extracted and ethanol precipitated. PCR for murine *Aldh1a1* gene promoter regions containing potentially active AhR binding sites was performed using the oligonucleotides: *Aldh1a1*-proximal 5′-CCTTTGTTCCGGAGTCTGTT-3′ (forward) and 5′- TTTACCAAGCCAAACCTGTG-3 (reverse); *Aldh1a1*-distal 5′-ATGGCTCATTGGCTAATCGT-3′ (forward) and 5′-GTGCAAGTGTGAGAGGAAGG-3′ (reverse). Diluted samples of total DNA were amplified as the input fractions. Negative controls were done in the absence of specific antibody. The amplified DNA was separated on 2.5 % agarose gels and visualized by ethidium bromide staining.

### Cell proliferation and cell cycle analyses

Cell proliferation was determined by quantifying the increase in cell numbers over time (up to 72 h) in B16F10 and sh-Aldh1a1 cell cultures. The number of cells attached to the plates was obtained after tripsinization and counting in a TC-10 automated cell counter (Bio-Rad). Flow cytometry was used to analyze cell cycle distribution. Cells were trypsinized, washed in PBS, fixed at 4 °C in 70 % cold ethanol and treated with RNase (10 mg/ml) for 30 min at 37 °C. DNA content per cell was determined in a Cytomics FC500 flow cytometer after staining with propidium iodide (50 μg/ml) for 15 min at RT in the dark. For cell cycle analysis, only signals from single cells were considered (10.000 cells/sample).

### Aldh1a1 activity

Aldh1a1 activity was measured using the Aldefluor reagent kit (StemCell Technologies) following the instructions of the manufacturer. In brief, cells were incubated for 60 min at 37 °C with the Aldefluor reagent in absence or presence of the Aldh1 inhibitor diethylamino-benzaldehyde (DEAB). After incubation, aliquots of 10^4^ cells were analyzed for Aldh1a1 activity in a Cytomics FC500 cytometer (Beckman Coulter). Non-viable cells were excluded from the analysis by propidium iodide staining. Enzymatic activity was normalized with respect to that measured in DEAB-treated cells.

### Clone formation

Clone formation was analyzed by platting 5×10^2^ or 10^3^ sh-AhR or sh-AhR + sh-Aldh1a1 cells in plain culture dishes for 5 days. Then, clones were gently washed in PBS and stained for 10 min with 0.5 % (w/v) crystal violet. Clones were counted and analyzed for cell spreading using the ImageJ software (version 1.45S). A qualitative scale was established to account for the appearance of unexpanded (compact clones with a dense cellularity), expanded (clones in which cells spread out and had reduced cell-cell interactions) or intermediate clones.

### Cell migration and invasion

Cell migration was analyzed in a Cell-R fluorescence microscope (Olympus, Tokyo, Japan) using 2-D wound healing assays as reported [21, 23]. The ability of B16F10 cell lines to invade through matrigel-coated transwells was determined using an Olympus FV1000 confocal microscope as described [56].

### Melanosphere formation

sh-AhR and sh-AhR + sh-Aldh1a1 cells were grown at 3×10^4^ cells/well in ultra-low adherence 24-well plates (Nunc, Thermo Fisher). After 24 h, melanospheres formed were analyzed with an Olympus Cell-R microscope. Sphere number and size were quantified using the ImageJ software (version 1.45S).

### Flow cytometry of side population (SP) and CD^+^ cells

Side population cells (SP) present in sh-AhR and sh-AhR + sh-Aldh1a1 cultures were first analyzed using published criteria [57] in a MoFlo-XDP equipment (Beckman-Coulter). To isolate SP cells, cultures were stained with 5 μg/ml Hoechst 33342 (Sigma-Aldrich) for 90 min at 37 °C, centrifuged and resuspended in HEPES-HBSS buffer containing 2 % FBS. Aliquots of cells were treated with 50 μM of the ABCG2 extrusion pump inhibitor fumitremorgin C (Sigma-Aldrich). Propidium iodide (10 nM) was used to discriminate dead cells. To quantify SP subpopulations, Hoechst 33342-labeled cells were stained for CD133^+^ (CD133-PE), CD44^+^ (CD44-PerCP) and CD29^+^ (CD29-FITC) during 30 min at 10 °C. To-Pro (0.1 μM) was used to discriminate dead cells from the analyses.

### Melanoma primary tumorigenesis and lung metastasis

Aliquots of 10^5^ exponentially growing sh-AhR and sh-AhR + sh-Aldh1a1 cells were injected subcutaneously in either flank of *AhR+/+* immunocompetent recipient mice and tumors allowed to grow for 15 days. Mice were killed and tumors recovered, weighted and measured with a caliper. Tumor volume was calculated using the formula: [length × width^2^ × 0.4]. For lung metastasis assays, 10^5^ cells were resuspended in 100 μl PBS and injected through the tail vein of *AhR+/+* immunocompetent recipient mice. After 21 days, mice were killed and their lungs extracted and analyzed for the presence of melanoma-derived metastatic nodules.

### In vivo imaging (IVIS)

The metastatic potential of sh-AhR and sh-AhR + sh-Aldh1a1 melanoma cells was also analyzed in vivo using the IVIS imaging system. Cells were further engineered to stably express the luciferase gene (LMP:sh-AhR:LUX and LMP:shAhR + shAldh1a1:LUX). C57BL6 albino mice at 8 weeks of age were injected through the tail vein with 10^6^ sh-AhR:LUC or sh-AhR + sh-Aldh1a1:LUC cells in 50 μl DMEM medium. Mice were analyzed for metastatic dissemination at the time of injection (0 days) and after 2, 7, 11, 14 and 21 days. At each time point, mice were anesthetized with isofluorane and injected i.p. with 30 mg firefly luciferin in 100 μl PBS. Mice were then quickly introduced into the IVIS equipment (Xenogen) and images collected in dorsal and ventral positions. At day 21, mice were killed and their organs photographed and fixed in buffered formalin for further analyses. The emission of luminescence was quantified as photons per second using the Living Image software (Xenogen).

### Statistical analyses

Data are shown as mean ± SD. Comparisons between experimental conditions was done using GraphPad Prism 6.0 software (GraphPad). The student’s *t* test was used to analyze differences between two experimental groups and ANOVA for the analyses of three or more groups. The Mann–Whitney non-parametric statistical method was used to compare rank variations between independent groups. Experiments were done in duplicate or triplicate in at least three cultures of each cell line.

## Additional file

Additional file 1: Figure S1. (A) AhR expression does not significantly affect *Aldh1a3* mRNA levels. Wild-type B16F10, sh-AhR and CA-AhR melanoma cells were analyzed for *Aldh1a3* expression by RT-qPCR using total RNA and specific oligonucleotides. RT-qPCR data were normalized by *Gapdh* expression and represented as 2-ΔΔCt. (B) AhR does not bind the murine *Aldh1a1* gene promoter under our experimental conditions. The same cell lines were analyzed for AhR binding to the murine *Aldh1a1* promoter by chromatin immunoprecipitation assays (ChIP) using the affinity purified AhR antibody (SA-210) (AhR Ab). A representative experiment for the proximal XRE sites is shown. Similar results were obtained for the distal XRE sites (see the Methods). Positive and negative controls include total DNA input (Input) and immunoprecipitations performed in presence of IgG, respectively. Determinations were done in duplicate in two different cell cultures. Data are shown as mean ± SE. (PPTX 941 kb)

## Abbreviations

Aldh: Aldehyde dehydrogenase
AhR: Dioxin receptor
A.U.: Arbitraty units
EMT: Epithelial-to-mesenchymal transition
FICZ: 6-formylindolo[3,2-b]carbazole
PBS: Phosphate buffered saline
RT-qPCR: Real-time reverse transcription-PCR
