# Supplementary material for: Dioxin receptor regulates aldehyde dehydrogenase to block melanoma tumorigenesis and metastasis
Source: Mol Cancer. 2015 Aug 5;14:148. doi: 10.1186/s12943-015-0419-9 (PMC4524442; doi:10.1186/s12943-015-0419-9)
Supplement: Additional file 1: Figure S1. — (A) AhR expression does not significantly affect Aldh1a3 mRNA levels. Wild-type B16F10, sh-AhR and CA-AhR melanoma cells were analyzed for Aldh1a3 expression by RT-qPCR using total RNA and specific oligonucleotides. RT-qPCR data were normalized by Gapdh expression and represented as 2-ΔΔCt. (B) AhR does not bind the murine Aldh1a1 gene promoter under our experimental conditions. The same cell lines were analyzed for AhR binding to the murine Aldh1a1 promoter by chromatin immunoprecipitation assays (ChIP) using the affinity purified AhR antibody (SA-210) (AhR Ab). A representative experiment for the proximal XRE sites is shown. Similar results were obtained for the distal XRE sites (see the Methods). Positive and negative controls include total DNA input (Input) and immunoprecipitations performed in presence of IgG, respectively. Determinations were done in duplicate in two different cell cultures. Data are shown as mean ± SE. (PPTX 941 kb) [file 12943_2015_419_MOESM1_ESM.pptx]

## Slide 1
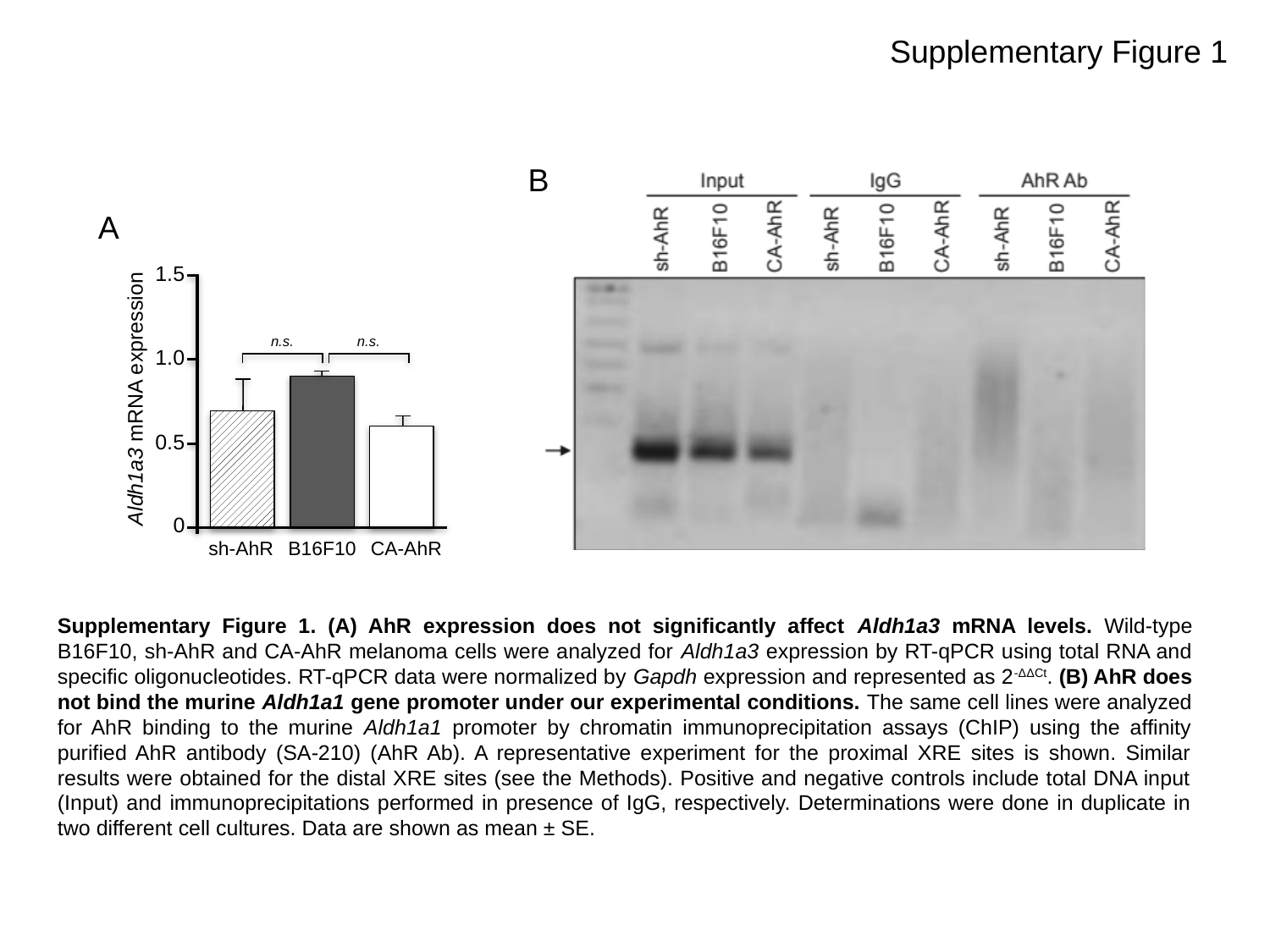

Supplementary Figure 1
B
A
1.5
n.s.
n.s.
1.0
Aldh1a3 mRNA expression
0.5
0
sh-AhR
B16F10
CA-AhR
Supplementary Figure 1. (A) AhR expression does not significantly affect Aldh1a3 mRNA levels. Wild-type B16F10, sh-AhR and CA-AhR melanoma cells were analyzed for Aldh1a3 expression by RT-qPCR using total RNA and specific oligonucleotides. RT-qPCR data were normalized by Gapdh expression and represented as 2-ΔΔCt. (B) AhR does not bind the murine Aldh1a1 gene promoter under our experimental conditions. The same cell lines were analyzed for AhR binding to the murine Aldh1a1 promoter by chromatin immunoprecipitation assays (ChIP) using the affinity purified AhR antibody (SA-210) (AhR Ab). A representative experiment for the proximal XRE sites is shown. Similar results were obtained for the distal XRE sites (see the Methods). Positive and negative controls include total DNA input (Input) and immunoprecipitations performed in presence of IgG, respectively. Determinations were done in duplicate in two different cell cultures. Data are shown as mean ± SE.
